# Engineering High-Performance Asphalt Binders and Mixtures Through Micro- and Nanocoke/Polymer Hybrid Modification

**DOI:** 10.3390/polym18161994

**Published:** 2026-08-16

**Authors:** Yerdos Ongarbayev, Muhammad Hashami, Yerbol Tileuberdi, Yerzhan Imanbayev, Ainur Zhambolova, Yernar Kanzharkan, Aliya Kenzhegaliyeva, Aksaule Kydyrali, Dinmukhamed Abdikhan, Talgar Serik

**Affiliations:** 1 Faculty of Chemistry and Chemical Technology, Farabi University, 71, Al-Farabi Ave., Almaty 050040, Kazakhstan; aksaule2014r@gmail.com (A.K.); dimash.abdixan02@gmail.com (D.A.); 2 Institute of Combustion Problems, 172, Bogenbai Batyr Str., Almaty 050012, Kazakhstan; er.tileuberdi@gmail.com (Y.T.); erzhan.imanbayev@mail.ru (Y.I.); zhambolova.ainur@mail.ru (A.Z.); erxank@mail.ru (Y.K.); aliakenzik@gmail.com (A.K.); talgarserik0@gmail.com (T.S.); 3 Department of Chemistry, Faculty of Education, Mirwais Khan Nika Zabul University, Qalat 4001, Afghanistan; mg.hashami2010@gmail.com; 4 Faculty of Natural Science and Geography, Abay Kazakh National Pedagogical University, 13, Dostyk Ave., Almaty 050010, Kazakhstan

**Keywords:** polymer-modified bitumen, nanocoke, hybrid nanocomposite modifiers, dynamic shear rheometer, pavement performance

## Abstract

Polymer modifiers and carbon-based materials have been widely proposed as potential asphalt additives to improve performance. However, limited studies have systematically compared micro- and nanocoke from different sources in combination with SBS and Elvaloy polymers or linked binder rheology with mixture performance. To address this gap, this study investigated the effects of micro- and nanodispersed petroleum and coal coke combined with SBS and Elvaloy polymers on the rheological behavior of asphalt binders and the performance of asphalt concrete mixtures. Rheological properties were evaluated by dynamic shear rheometer (DSR) testing, and asphalt concrete mixtures were tested for compressive strength and crack resistance. The modified systems were successfully used to obtain commercial polymer-modified bitumen grades: BMP 70/100, BMP 50/70 and BMP 35/50. With 1 wt.% micro coal coke and 0.1 wt.% SBS modification, a softening point of 73.7 °C and a penetration of 22.3 × 0.1 mm was observed. Micro coal coke–SBS system showed the highest compressive strength at 20 °C (3.29 MPa), while the largest crack resistance (4.39 MPa) and the best high-temperature mixture strength (0.90 MPa at 50 °C) were obtained when 0.5 wt.% nanocoke and 0.5 wt.% Elvaloy are used. These findings demonstrate that hybrid coke/polymer modification is an effective approach for enhancing asphalt performance, with micro coal coke–SBS systems providing the highest stiffness and rutting resistance, while nanocoke–Elvaloy systems delivered superior crack resistance and overall performance balance.

## 1. Introduction

Bitumen is known as the most common binder used in flexible pavement systems, but its inherent rheological characteristics can strongly degrade pavement durability and service life. A major problem is rutting, caused by the accumulation of permanent deformation due to repeated traffic loading, especially at high temperatures. This behavior is primarily associated with the dominant viscous response of unmodified bitumen, typically characterized by high phase angle values and a low complex shear modulus. In addition, bitumen is very sensitive to temperature, meaning that it melts too easily at high temperatures and hardens too quickly at low temperatures, causing fatigue and thermal cracking [1]. Environmental exposure, including oxidation and moisture exposure, further promotes the degradation of binders and decreases adhesion between aggregates and the binder, which ultimately affects long-term pavement performance [2]. Therefore, the development of advanced modification strategies is necessary to achieve a more favorable viscoelastic balance and improve the durability of asphalt binders [3].

It is well known that polymer modifiers can improve the properties of bitumen such as its elasticity, stiffness and temperature susceptibility [4]. Teltayev et al. [5] demonstrated that polymer-modified binders exhibit significantly improved low-temperature crack resistance and high-temperature rutting resistance owing to their enhanced viscoelastic balance. Jasso et al. [6] compared SBS-, Elvaloy-, and polyphosphoric acid-modified binders and reported that SBS effectively improves elasticity through the formation of a physically cross-linked polymer network, whereas Elvaloy enhances the storage stability by forming chemical bonds with bitumen components. More recently, Yan et al. [7] comprehensively reviewed the modification mechanisms of SBS and highlighted its ability to reduce the phase angle, increase the complex modulus, and improve the rutting and fatigue performance of asphalt mixtures. The long-term stability of polymer-modified systems still remains a concern, especially for SBS, which is subject to thermolysis during the storage at high temperature (160–180 °C), causing the degradation of its mechanical properties with the formation of chain scission [8,9]. To overcome this limitation, Geçkil [10] demonstrated that reactive Elvaloy polymer improves the chemical compatibility, microstructural homogeneity, and storage stability of modified binders through covalent bonding with asphalt constituents. These studies demonstrate that polymer modification can substantially improve the rheological and mechanical performance of asphalt; however, most investigations have focused on individual polymer systems, while the synergistic effects of combining different polymer chemistries with carbon-based modifiers remain insufficiently explored [11,12].

Other modification approaches, including epoxy modification, ozonation, and mechanochemical activation, have also been investigated to tailor the molecular and structural characteristics of bitumen [13,14]. These treatments can alter the colloidal structure and thermoplastic behavior of bitumen, potentially improving the durability of asphalt materials [15,16]. Importantly, recent nanoscale studies have shown that the thermal history of bitumen and its preparation conditions can significantly modify the surface morphology as well as local mechanical and chemical characteristics, emphasizing the need to carefully control the thermal treatment when evaluating the microstructural effects of different modification strategies [17]. Such thermal-history-dependent structural changes are particularly relevant to modified binders because the processing conditions may influence the observed dispersion, interfacial interactions, and microstructural organization of modifier–bitumen systems.

Carbonaceous materials have emerged as promising multifunctional modifiers for improving the mechanical and high-temperature performance of asphalt binders. High-temperature performance and structural integrity are enhanced using these materials, such as carbon black, graphene, carbon nanotubes and coke-based particles. For example, mechanochemically activated microcoke has been reported to enhance high-temperature rheological characteristics and make the product more resistant to deformation [18,19]. Biochar-modified binders exhibit enhanced stiffness and microstructural uniformity, with optimized compositions achieving the best mechanical performance [20]. These materials are used as reinforcing filler materials to increase the load transfer component of the binder, reduce the molecular mobility, and improve its viscoelastic properties and resistance to deformation [21]. The functional and oxidation resistance characteristics of bitumen are further improved by advanced chemical treatments like the use of ozonation, which results in better long-term durability [22]. Collectively these findings indicate that the blending of the polymer and carbon modifiers is a potential route to prepare high-quality asphalt binders that possess excellent rheological and mechanical properties [23]. Most of the recent studies have been centered on the measurement of binder-level rheological properties or measurement of mixture-level mechanical performance, with limited efforts dedicated to establishing a direct correlation between these two scales [21]. There is a lack of systematic comparison of the microdispersed and nanodispersed coke materials, especially as it relates to their reinforcement mechanisms and effectiveness [23]. A comparative study of these two types of coke is also limited, due to their different physico-chemical properties and interaction mechanisms with bitumen. In addition, systematic comparisons of microdispersed and nanodispersed coke, particularly with respect to coke origin and polymer chemistry, remain limited. The effects of petroleum versus coal coke and SBS versus Elvaloy on the resulting structure–property relationships have therefore not been sufficiently clarified.

To address these gaps, this study systematically investigates hybrid coke/polymer modification by considering three key variables: coke particle size (micro versus nano), coke origin (petroleum versus coal), and polymer chemistry (SBS versus Elvaloy). The study evaluates both binder-level rheological behavior using dynamic shear rheometry and asphalt-mixture mechanical performance, including compressive strength and crack resistance. By integrating these two scales, the work aims to identify the most effective coke/polymer combinations for achieving an appropriate balance among stiffness, elasticity, rutting resistance, and crack resistance. This comparative framework provides a clearer understanding of how the coke particle size, source, and polymer type jointly influence asphalt performance and supports the development of high-performance modified asphalt binders and mixtures.

## 2. Materials and Methods

### 2.1. Materials

The materials used in the study were petroleum and coal coke samples manufactured by the Pavlodar Petrochemical Plant LLC (Pavlodar, Kazakhstan), bituminous materials at bitumen grade BND 100/130 supplied by “Asphaltbeton 1” LLC (Almaty, Kazakhstan), polymer modifier styrene–butadiene–styrene (SBS) copolymer and Elvaloy reactive polymer.

Table 1 presents some physico-chemical properties of the petroleum and coal coke samples. The petroleum coke sample exhibited low moisture (0.2 wt.%) and ash content (0.1 wt.%), which can indicate its high carbon purity and thermal stability. Coal coke is characterized by a relatively low sulfur content (1.1%).

Table 2 presents the physico-mechanical properties of the BND 100/130 bitumen. Bitumen is characterized by a high dynamic viscosity at 135–352 mm^2^/s, which confirms the completeness of the mixing of the bitumen binder with inert materials. The flash point of the bitumen is 282 °C, the brittleness temperature according to Fraas is −24 °C, and the paraffin content is 0.4 wt.%.

The properties of SBS and Elvaloy polymers are presented in Table 3. As can be seen from the table, the polymers have the same density of 0.94 g/cm^3^. The melt index of Elvaloy is 10 times higher than that of SBS. This indicator indicates how easily a thermoplastic material can flow under a given pressure and temperature. Both modifiers can be used in the temperature range from 72 to 280 °C. Elvaloy is characterized by a low volatile matter content (0.04 wt.%) and ash content (0.2 wt.%).

### 2.2. Production of Micro- and Nanocoke Samples

The micro- and nanodispersed coke powders used in this study were prepared by mechanochemical activation and controlled dispersion processes, which allow a reduction in the particle size and an increase in the surface activity of coke particles, consequently resulting in increased interaction with the bitumen matrix [19].

Microdispersed coke powders were produced via mechanochemical activation using a GT Planetary mill (Powteq, Beijing Grinder Instrument Co., Ltd., Beijing, China). Grinding was conducted at coke-to-steel ball mass ratios of 1:1 and 1:2, with a rotational speed of 1200 rpm and milling durations of 20, 40, and 60 min. This approach enabled controlled particle size reduction and structural activation of the coke materials.

Nanodispersed coke samples materials were obtained using chemical vapor deposition (CVD). The CVD treatment of coke samples was carried out at a temperature of 1000–1200 °C in an inert atmosphere. Starting from room temperature, the samples were heated to the target temperatures at a rate of 10 °C/min. An inert gas flow was supplied to the reaction zone at a rate of 1 L/min. After reaching the target temperature, the samples were held at this temperature for 30 min. The samples were then cooled to room temperature at a rate of 10 °C/min.

Morphological features and surface textures of the micro- and nanocoke powders were examined using a JSM-6490LA scanning electron microscope (SEM) (JEOL Ltd., Tokyo, Japan).

### 2.3. Preparation of Modified Binders

**Figure 1:**
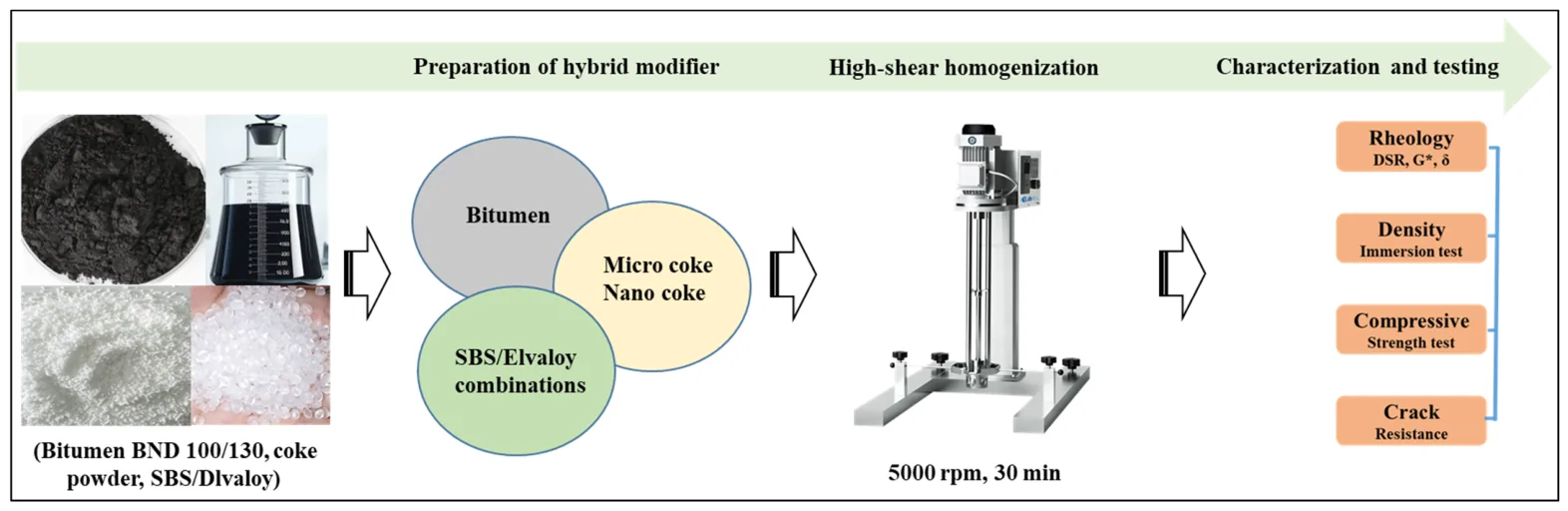
Flowchart of the preparation and testing procedure for coke- and polymer-modified bitumen binders, from raw material selection (bitumen BND 100/130, coke powder, SBS/Elvaloy) to the characterization methods used to evaluate the binders (rheology, density, compressive strength, and crack resistance).

The base bituminous material was modified with micro- and nanodispersed coke powder at concentrations of 0.1, 0.5 and 1 wt.%. The process was performed using a propeller mixer operating at 750 rpm at a temperature of 160 °C for 1 h to ensure the uniform dispersion of the coke particles. Polymer–bitumen binders were prepared by introducing coke powders in a range of 0.1–1 wt.% to a bitumen system via a propeller mixer at 700 rpm and 165 °C for 1 h. The mixtures were then homogenized at high shear rate (5000 rpm) for 30 min to ensure a stable and homogeneous structure. The polymer modifiers SBS and Elvaloy were added between 0.1 and 1.5 wt.% depending on the formulation. The combination of controlled mixing temperature, high-shear homogenization, and mixing duration was selected to promote a uniform distribution of the coke particles throughout the bitumen matrix before testing. The complete preparation workflow, from raw material selection to the characterization stage, is summarized in Figure 1.

### 2.4. Preparation of Asphalt Concrete Mixtures

To produce modified asphalt concrete mixture samples, the following starting materials were used: crushed stone (10–20 mm and 5–10 mm fractions), sand from crushed stone screenings (1–5 mm fraction), activated mineral powder (MP-1), bitumen grade BND 100/130 from “Asphaltbeton 1” LLC, micro- (5–20 µm) and nanodispersed (less than 1 µm) petroleum and coal coke powders, and SBS and Elvaloy polymer additives.

The materials were mixed using the hot mixing technology. Crushed stone, crushed sand, and coke powders were heated in a drying oven to a temperature of 160–170 °C, the bitumen binder to 145 °C, and the mineral powder was added cold. The mixture was mixed in two stages. In the first stage, crushed stone, crushed sand, coke powder, and mineral powder were dry-mixed. In the second stage, hot bitumen was added (wet mixing) while continuously mixing the materials. Mixing was continued until homogeneity was achieved. Homogeneity was assessed visually by the degree of coating of the grains with the bitumen binder. The temperature of the finished asphalt concrete mixture was 150–160 °C. Cylindrical specimens 71.4 mm in height and diameter were produced under a constant load of 40 MPa in accordance with ST RK 1218-2003. After molding, the specimens were kept in an air-dry state for 24 h.

Two types of compositions with the addition of modified bitumen were prepared for testing. The composition of the mixtures was as follows: 10–20 mm crushed stone—23 mass %; 5–10 mm crushed stone—24.5 mass %, 1–5 mm screenings—40 mass %, mineral powder—7 mass %, bitumen binder modified with micro- or nanodispersed coke powder and polymer—5.5 mass %.

### 2.5. Test Methods

#### 2.5.1. Testing of Conventional Properties of Asphalt Binders

The physico-mechanical properties of the asphalt binders were determined in accordance with the current standards of the Republic of Kazakhstan. Penetration was determined according to ST RK 1226-2003 using a PN-10B apparatus (JSC BSDB “Neftekhimavtomatika”, Ufa, Russia). The softening point was determined according to ST RK 1227-2003 using a KISH-20 apparatus (JSC BSDB “Neftekhimavtomatika”, Ufa, Russia).

#### 2.5.2. Rheological Testing

The rheological properties of the prepared binders were measured in accordance with the European standard DIN EN 14770:2023, with the help of an Anton Paar SmartPave 102e dynamic shear rheometer (DSR, Anton Paar GmbH, Graz, Austria). The complex shear modulus (G*) and phase angle (δ) were obtained at controlled strain within the linear viscoelastic region (about 0.1%) at the temperature range from 0 up to 100 °C, using the parallel plates geometry, with a 1.0–2.0 mm gap between plates and with the diameter of 25 mm for low-temperature and 8 mm for high-temperature tests. The shear stress, shear strain, storage modulus (G′), loss modulus (G″), complex modulus (G* = √(G′^2^ + G″^2^)), and phase angle (δ = arctan(G″/G′)) were measured and recorded by the instrument under sinusoidal shear stress at a constant angular frequency of 10 rad/s (1.59 Hz). The data obtained from each temperature step were used to build temperature-dependent rheological curves and to analyze the viscoelastic properties of the binders [24]. Measurements were performed automatically using the loop function of the RheoCompass software. Twenty data points were recorded at each temperature point. Before measurement, the sample was held until thermal equilibrium was reached. The tests were conducted within the linear region of the viscoelastic behavior of the material.

After short-term aging (heat treatment at 163 °C for 85 min) in a Rolling Thin Film Oven Test (RTFOT), the samples were tested in a temperature range from 40 to 82 °C in 6 °C increments. Based on the complex shear modulus and phase shift angle, the shear resistance parameter G*/sinδ was calculated.

To evaluate the low-temperature properties after long-term aging (heat treatment at 163 °C for 225 min), tests were conducted in a Pressure Aging Vessel (PAV) system over a temperature range of 25 to 4 °C with a 3 °C increment. The complex shear modulus (G *) and phase shift angle (δ) were determined from the measurements, and the fatigue resistance parameter G*⋅sinδ was then calculated.

#### 2.5.3. Density

The physico-mechanical properties of asphalt concrete mixtures were determined using standardized procedures. The average density was determined by first weighing the specimens in air and then placing them in water at 20 ± 2 °C for 30 min to a depth of not less than 20 mm above the surface of the sample. Samples were then weighed in water (without air entrapment), dried with a soft cloth, and reweighed in air. The average density (*ρ_m_*, g/cm^3^) was calculated using the following formula:

(1) $$\rho_{m}=\frac{g\rho^{w}}{g_{2}-g_{1}}$$

where *g* is the mass of the sample suspended in air, *ρ^w^* is the density of water (*ρ^w^* = 1 g/cm^3^), the mass in water is (*g*_1_), and the mass after soaking and reweighing in air is (*g*_2_).

#### 2.5.4. Compressive Strength

The compressive strength of the specimen was calculated by applying increasing load until the specimen broke in a controlled setting. All samples were thermostated at 20 ± 2 °C before testing, and samples from hot mixtures were placed in water at the same temperature for 1 h prior to testing. The test was conducted using a press with a platen speed of 3.0 ± 0.3 mm/min. The specimen was placed in the middle of the lower platen, and the upper platen was set at 1.5–2 mm from the sample surface prior to loading. The maximum force recorded was taken as the failure load, and the compressive strength (*Rc*, MPa) was calculated using the ratio of the ultimate load (*P*) to the initial cross-sectional area (*F*), with a conversion factor of 10^−2^. The reported value corresponds to the average of three specimens. The compressive strength *Rc*, in MPa, is calculated using the formula:

(2) $$Rc=\frac{P}{F}10^{-2},$$

where *P* is the ultimate tensile strength in N, and *F* is the original cross-sectional area of the specimen in cm^2^; then, 10^−2^ is the conversion factor to convert the stress in N/cm^2^ to the stress in MPa.

#### 2.5.5. Crack Resistance

Crack resistance was measured using the indirect tensile strength (splitting) method, which measures the force needed to break the specimen along its generatrix. All samples were refrigerated for at least 1 h in an ice-water solution before testing. The test was performed using a press operating at a constant platen speed of 50 ± 2 mm/min. The specimen was placed horizontally on the lower platen and load applied until failure, the maximum load recorded. The expression for calculating crack toughness (*Rp*, MPa) is as follows:

(3) $$Rp=\frac{P}{hd}10^{-2},$$

where *P* is the ultimate load (N), *h* is the specimen height (cm), *d* is the specimen diameter (cm), and 10^−2^ is the conversion factor (MPa). The final value was obtained as the average of three measurements, rounded to one decimal place.

## 3. Results

### 3.1. Micro- and Nanocoke Morphological Characterization

**Figure 2:**
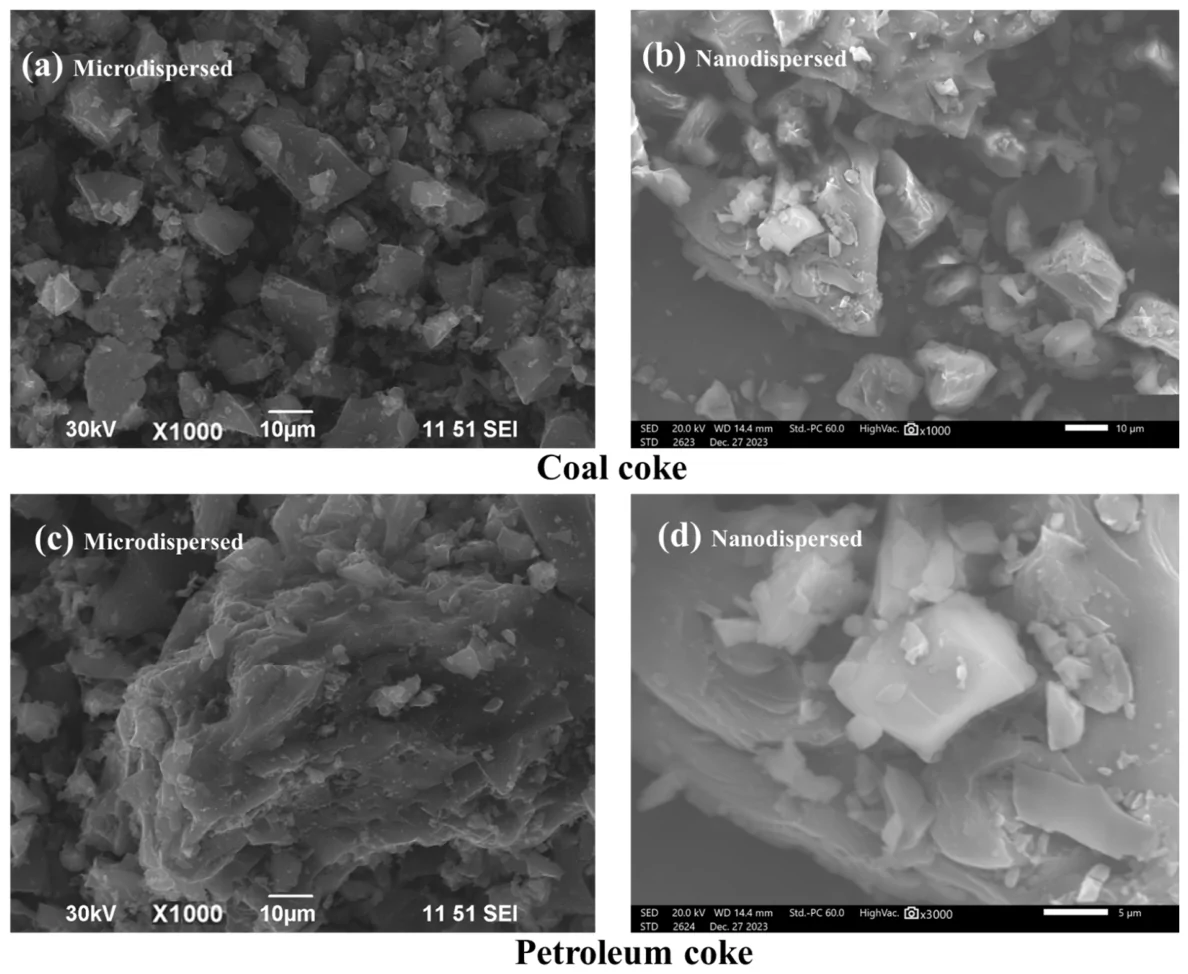
SEM images of the coke powders used for bitumen modification: (**a**) microdispersed coal coke, (**b**) nanodispersed coal coke, (**c**) microdispersed petroleum coke, and (**d**) nanodispersed petroleum coke. The images illustrate the morphological and particle-size differences between the micro- and nanodispersed coke modifiers.

The performance of modified asphalt binders depends as much on the chemical composition of the binder as it does on the morphology, particle size, surface texture and dispersion of the modifier. The morphological characteristics determine the absorption of light aromatic fractions, the adhesion at the interface between particles and bituminous matrix, the efficiency of stress transfer and the formation of a reinforced internal structure, which governs the interaction between the particles and the bituminous matrix. SEM images of the micro- and nanodispersed coal and petroleum coke samples used in this study are shown in Figure 2a–d.

The microdispersed coal coke has an irregular angular shape with a rough heterogeneous surface (Figure 2a). Particles are mostly in the 2–15 μm range, with some agglomerates up to 20 μm, consistent with the fractured porous morphology typical of coal-derived cokes [25]. The angular geometry and rough surface texture are expected to increase the mechanical interlocking and restrict the molecular mobility within the asphalt matrix, which is consistent with the micro coal coke-modified binders showing the lowest penetration values and highest softening temperatures among the modifiers tested.

The nanodispersed coal coke (Figure 2b) shows a significantly smaller and more uniform particle size, with the majority below 1 μm, while retaining the angular shape and rough surface of the microdispersed form. The higher surface area is expected to improve the stress-transfer efficiency and interfacial adhesion with the bitumen [26], which may explain the favorable stiffness–elasticity balance observed for nanocoal coke-modified binders in the rheological analysis.

Microdispersed petroleum coke (Figure 2c) has a markedly different, layered, lamellar morphology with smoother surfaces and particle sizes of about 5–20 μm, characteristic of the anisotropic carbon structure formed during delayed coking [27]. The reduced surface roughness and fewer sharp edges likely explain the weaker mechanical interlocking and the correspondingly higher penetration and lower softening point values observed for petroleum coke-modified binders relative to coal coke.

The nanodispersed petroleum coke (Figure 2d) retains the lamellar structure but as thin nanometer-scale platelets below 1 μm, providing a much larger exposed surface area for interaction with the bitumen matrix. This morphology is expected to improve the dispersion and interfacial contact during high-shear mixing, consistent with reports for other nanostructured carbon modifiers [28], and likely contributes to the enhanced rheological performance observed when combined with Elvaloy and SBS polymers.

The enhanced dispersion of the petroleum coke nanoparticles is likely to contribute to the rheological enhancement, especially if used in combination with Elvaloy and SBS polymers. The morphological trends described above are discussed jointly with the corresponding penetration, softening point, and rheological results in Section 3.2, Section 3.3 and Section 3.4, where the correlation between the coke microstructure and macroscopic binder performance is examined in more detail. It should be noted that the morphological characterization presented here was performed on the raw coke particles prior to incorporation into bitumen. While the observed particle geometry, size and surface texture provide a reasonable basis for interpreting the trends in penetration, softening point and rheological behavior, direct visualization of the coke–bitumen interface (e.g., via SEM/AFM of the blended binder) and chemical characterization (e.g., FTIR) were beyond the scope of this study and are recommended for future work to further verify the proposed interaction mechanisms.

### 3.2. Modified Polymer–Bitumen Binder Conventional Properties

The conventional properties of asphalt binders, particularly the penetration and softening point, are widely used indicators of binder consistency and temperature susceptibility. Lower penetration values indicate increased stiffness and resistance to permanent deformation, whereas higher softening points reflect improved thermal stability and rutting resistance at elevated service temperatures. The incorporation of coke particles together with polymer modifiers significantly altered these properties, demonstrating the effectiveness of both coke type and polymer type in tailoring binder performance.

The results presented in Table 4 reveal that the addition of 1 wt.% microdispersed coke together with SBS or Elvaloy polymers substantially modified the consistency of the BND 100/130 base bitumen. The most pronounced effect was observed for microdispersed coal coke combined with 0.1 wt.% SBS, which produced a binder with a softening point of 73.7 °C and a penetration of only 22.3 (0.1 mm). Compared with binders containing microdispersed petroleum coke, whose penetration values ranged between 57.7 and 70.7 (0.1 mm), coal coke-modified binders exhibited significantly lower penetration values and higher softening temperatures, indicating the development of a much stiffer binder structure. This behavior suggests stronger interactions between coal coke particles and the bituminous matrix.

Comparing the two polymers shows different modification mechanisms. SBS-modified binders tended to have lower penetration values than the Elvaloy-modified systems in similar coke content systems. In the case of microdispersed coal coke, for instance, the penetration was raised from 22.3 to 60.3 (0.1 mm) when SBS was substituted with Elvaloy at 0.1 wt.% polymer content. This observation suggests that the stiffening effect and forming elastic network are more effective when using SBS. SBS provides an uninterrupted elastomeric phase in the asphalt matrix, enhancing the elasticity, and it helps to increase the resistance to rutting and permanent deformation. This phenomenon has been observed with SBS-modified binders where the polymer forms a three-dimensional network with the binder, which significantly enhances the binder stiffness and/or high-temperature performance [29,30].

Elvaloy-modified binders, on the other hand, had slightly higher penetration values and moderate softening temperatures, indicating a more moderated modification effect. The reactive terpolymer nature of Elvaloy encourages chemical bonding with the constituents of the asphalt, resulting in superior compatibility and less brittle, but still stiff, product. As a result, the use of Elvaloy-modified binders is usually correlated to better crack resistance and durability, especially under thermal and fatigue loadings [31,32]. These findings corroborate this finding, as Elvaloy binders sustained penetration values between the desirable range of 60 and 76.7 (0.1 mm), whereas the highly stiff SBS-modified coal coke systems did not.

The softening point of the coal coke-modified binders decreased with the increase in polymer, as its content increased from 0.1 to 1 wt.%. For instance, the softening point of the coal coke–SBS binder decreased from 73.7 °C at 0.1 wt.% SBS to 51.1 °C at 1 wt.% SBS, while the penetration increased from 22.3 to 68.0 (0.1 mm). The same effect was seen with coal coke–Elvaloy systems. This trend indicates that an over-addition of polymer may affect the interaction network of coke and bitumen and decrease the reinforcing effect of the coke particles. Some previous investigations have reported the existence of optimum polymer concentrations, where compatibility problems or phase rearrangements could reduce the stiffness gain [33].

In a specification aspect, the microdispersed coke systems were able to produce several commercial grade polymer-modified binders. Petroleum coke containing 0.1 wt.% SBS or Elvaloy fulfilled the requirements of BMP 70/100, while coal coke mixed with 0.5 wt.% SBS met the requirements for BMP 35/50 and with 0.5 wt.% Elvaloy, the requirements for BMP 50/70. These findings further validate the higher stiffening potential of coal coke than that of petroleum coke.

The results presented in Table 5 for the nanodispersed coke-modified binders show a contrasting modification behavior. Overall, the penetration of the nanocoke-containing binders ranged between 62.0 and 71.0 (0.1 mm), and the softening temperatures ranged between 48.9 and 63.1 °C, which is less pronounced than the reduction in penetration in the microdispersed coal coke systems, but is enough to satisfy the BMP 70/100 requirements. This means that when coke is dispersed to nanodimensions it offers a better stiffness–flexibility relationship.

The improved performance of nanocoke can be attributed to its significantly higher specific surface area and more uniform dispersion within the bitumen matrix. The nanoscale particles create a larger interfacial contact area with asphalt components, improving the stress transfer and reinforcing the efficiency without excessively restricting the molecular mobility. Consequently, the binder benefits from enhanced thermal stability while retaining sufficient flexibility to resist cracking. Similar observations have been reported for nanostructured carbon additives and nanofillers, which improve binder performance through microstructural reinforcement rather than excessive hardening [31].

At a nanocoke content of 0.5 wt.% and polymer content of 0.5 wt.%, both the petroleum and coal coke systems exhibited nearly identical properties. The softening points ranged from 62.0 to 63.1 °C, while the penetration values remained between 70 and 71 (0.1 mm), satisfying the requirements of BMP 70/100. Unlike the microdispersed systems, the difference between petroleum and coal coke became much less pronounced at the nanoscale, indicating that particle size plays a dominant role in determining the modification efficiency. The enhanced dispersion of nanocoke likely compensates for compositional differences between the two coke types.

### 3.3. Modified Polymer–Bitumen Binders’ Rheological Properties

**Figure 3:**
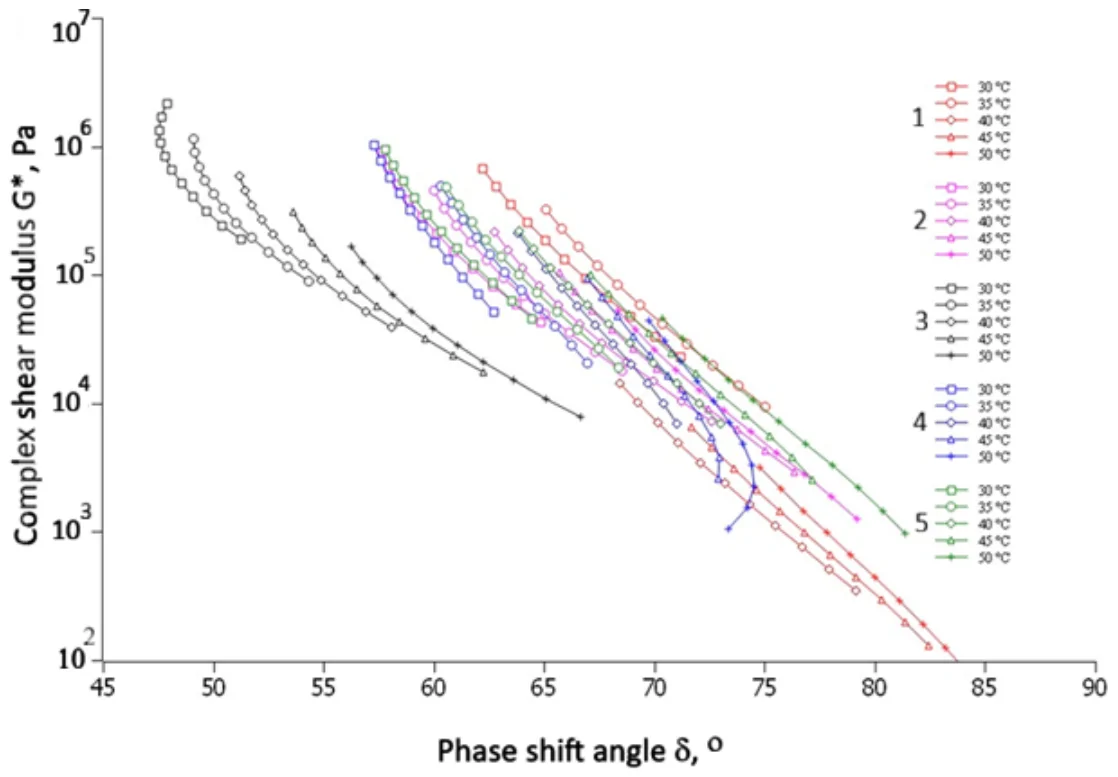
Black diagrams showing the relationship between complex shear modulus (G*) and phase angle (δ) for BND 100/130 bitumen and binders modified with microdispersed petroleum and coal coke combined with SBS or Elvaloy polymers at 30–50 °C: 1—BND 100/130 bitumen; bitumen-modified: 2—1 wt. % coal coke micropowder and 0.5 wt. % Elvaloy, 3—1 wt. % coal coke micropowder and 0.5 wt. % SBS, 4—1 wt. % petroleum coke micropowder and 0.1 wt. % SBS, 5—1 wt. % petroleum coke micropowder and 0.1 wt. % Elvaloy.

**Figure 4:**
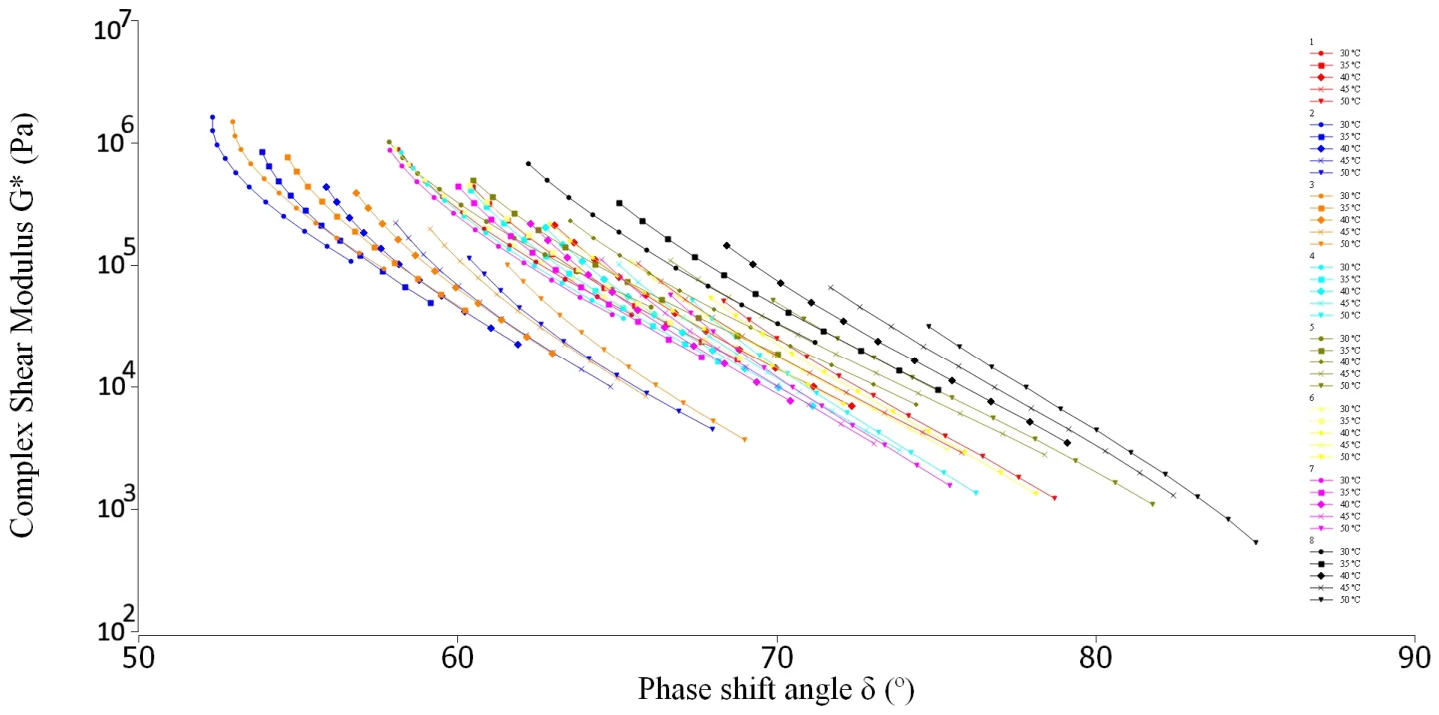
Black diagrams showing the relationship between complex shear modulus (G*) and phase angle (δ) for BND 100/130 bitumen and binders modified with coke nanopowders and Elvaloy polymer at 30–50 °C: 1—0.5% polymer and 0.5% coal coke nanopowder, 2—0.5% polymer and 0.5% petroleum coke nanopowder, 3—0.5% polymer and 0.1% petroleum coke nanopowder, 4—1% polymer and 0.1% petroleum coke nanopowder, 5—0.1% polymer and 0.1% petroleum coke nanopowder, 6—0.5% polymer and 0.1% petroleum coke nanopowder, 7—1% polymer and 0.1% coal coke nanopowder, 8—BND 100/130 bitumen.

**Figure 5:**
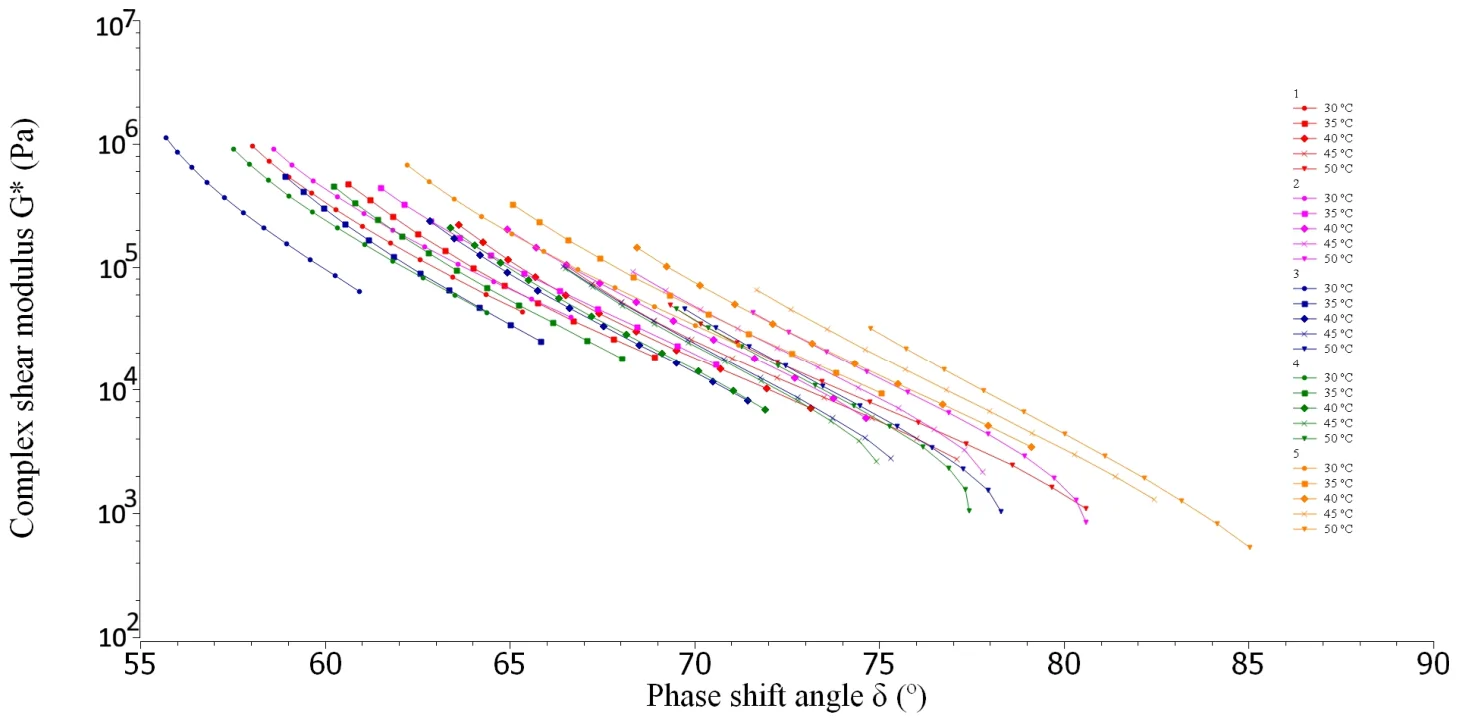
Black diagrams showing the relationship between complex shear modulus (G*) and phase angle (δ) for BND 100/130 bitumen and binders modified with coke nanopowders and SBS polymer at 30–50 °C: 1—1% polymer and 0.1% petroleum coke nanopowder, 2—0.1% polymer and 0.1% petroleum coke nanopowder, 3—0.5% polymer and 0.1% petroleum coke nanopowder, 4—0.5% polymer and 0.5% petroleum coke nanopowder, 5–BND 100/130 bitumen.

The complex shear modulus, which represents the total resistance of a material to deformation under oscillatory loading, is expressed as G* = √(G’^2^ + G″^2^), where G’ and G″ are the storage and loss moduli, respectively. Figure 3, Figure 4 and Figure 5 present the Black diagrams of the unmodified and modified binders, illustrating the relationship between the complex shear modulus (G*) and phase angle (δ) over the temperature range of 30–50 °C. The phase angle is defined as δ = tan^−1^(G″/G’) and reflects the relative contribution of viscous and elastic behavior. Lower phase angle values indicate a more elastic response, whereas higher values indicate a higher viscous component. Therefore, an increase in G* accompanied by a reduction in δ is generally associated with improved rutting resistance and enhanced high-temperature pavement performance [34].

The inclusion of microdispersed coke and polymer modifiers greatly enhanced the rheological properties of the base bitumen BND 100/130 (Figure 3). The primary binder had a phase angle of 75–85° and a low value of the complex shear modulus (10^2^–10^3^ Pa), which is a characteristic of viscous flow behavior and high susceptibility to temperature-induced deformation. On the contrary, all of the modified binders exhibited significantly high |G*| values and low phase angles, which indicated the improved elastic properties and thermal stability of the modified binders. The greatest enhancement was noted in the binder with 1 wt.% microdispersed coal coke and 0.5 wt.% SBS polymer, with phase angles of about 55–65° and moduli up to 10^4^–10^5^ Pa. This corresponds to a twofold higher modulus than the base bitumen, which is a very high level of resistance to shear deformation. The phase angle of the binder modified with coal coke and Elvaloy polymer was in the intermediate range (65–75°), and the lower modulus was ~10^3^–10^4^ Pa. The phase angles of the petroleum coke-modified binders varied in the range 70–80°, and the modulus values were lower, which suggested a lower structuring effect. The results show that the microdispersed coal coke is more effective than petroleum coke in increasing the stiffness of the binder, and SBS has a stronger elastic network than Elvaloy, which is better for resisting permanent deformation [35]. This leftward shift of the coal coke–SBS formulation relative to the other binders indicates a broader temperature range over which the material retains a low phase angle and high complex modulus. This behavior results from the combined reinforcing action of the angular high-surface-area coal coke particles, which restrict the molecular mobility within the bitumen matrix, and the continuous elastomeric network formed by SBS, which imparts elastic recovery. The synergy between these two mechanisms produces a more temperature-stable viscoelastic response than either component alone would provide, explaining why this formulation exhibited the lowest phase angles and highest moduli across the tested temperature range [36].

Figure 4 shows the rheological properties of binders modified with nanodispersed coke and Elvaloy polymer. Similar to Figure 3, all formulations exhibited the typical inverse relationship between G* and δ, whereby increasing temperature caused a decrease in the modulus and an increase in the phase angle. The nanocoke-modified binders showed a much narrower range of rheological properties than the microcoke systems, indicating more uniform reinforcement. All modified binders moved towards the left of the diagram to lower phase angles and higher moduli, while the original bitumen stayed at the right side of the diagram with phase angles up to about 85°. The formulations with 0.5 wt.% Elvaloy and 0.1–0.5 wt.% nanopowder of petroleum coke also showed the largest shift to the left, with phase angles below 55° and the modulus values above 10^4^–10^5^ Pa. This behavior shows increased elastic recovery and increased resistance to rutting. Binders with coal coke nanopowder, on the other hand, exhibited slightly higher phase angles at similar modulus values, suggesting a slightly lower elastic component. The findings indicate that the interaction between the petroleum coke nanoparticles and the Elvaloy-modified asphalt matrix is more efficient, resulting in a stronger and more stable microstructure. The same findings have been made for PMB with finely dispersed reinforcing particles [37]. The improved performance is explained by the high surface area of the nanoparticles, which increases the interactions between the nanoparticles and the asphalt constituents and hence the ability to transfer stress across the binder matrix [36].

The Black diagrams of binders modified with SBS polymer and petroleum coke nanopowder are shown in Figure 5. The SBS-modified binders demonstrated a larger shift towards lower phase angles when compared to the Elvaloy systems, further evidence that the SBS network contributes more to the elastic component. Formulations with 0.5 wt. % SBS and 0.1 wt. % petroleum coke nanopowder exhibited the highest elastic response of all nanocoke-modified formulations (phase angle range = 55.5–78°; modulus = 10^5^–10^6^ Pa at lower temperatures). In comparing the phase angles of the different formulations at the same modulus, this was a consistently lower angle, which corresponded to higher elastic recovery and higher resistance to permanent deformation. The binders with 0.5 wt.% SBS and 0.5 wt.% nanocoke and 1 wt.% SBS and 0.1 wt.% nanocoke showed almost the same rheological curves, indicating that the higher amount of nanocoke can be counterbalanced by a higher amount of SBS in order to achieve comparable performance. On the other hand, the sample with only 0.1 wt.% SBS and 0.1 wt.% nanocoke showed the highest phase angles among the modified samples, which means the formation of continuous elastic network was limited by the insufficient polymer content. The increase in elasticity with increasing SBS content is observed and is consistent with the known properties of SBS, which allows the creation of a three-dimensional polymer network in the asphalt matrix and thus enhances elastic recovery and prevents rutting [38]. The improved rheological properties of SBS-modified binders has also been extensively attributed to the enhanced polymer–bitumen compatibility and network stabilization effects provided by the SBS [39].

### 3.4. Modified Polymer–Bitumen Binders Rheological Properties After Aging

An analysis of the shear and fatigue resistance of modified BND 100/130 bitumen after short-term (RTFOT) and long-term (PAV) aging was carried out in accordance with the design principles of Superpave asphalt concrete mixtures (ST RK 4034-2025).

**Figure 6:**
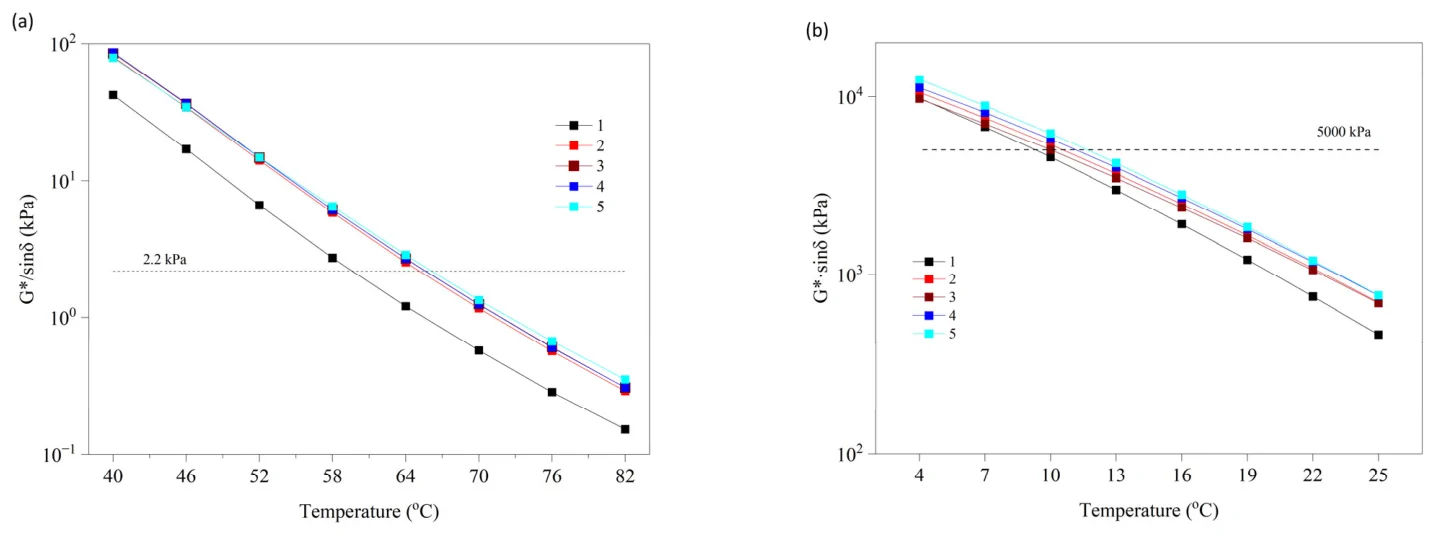
Dependences of shear (**a**) and fatigue (**b**) resistance of bitumen binders after short-term and long-term aging on temperature: 1—BND 100/130 bitumen, 2—with the addition of 0.1% Elvaloy and 1% petroleum coke micropowder, 3—with the addition of 0.1% SBS and 1% petroleum coke micropowder, 4—with the addition of 0.5% SBS and 1% coal coke micropowder, 5—with the addition of 0.1% Elvaloy and 1% coal coke micropowder.

Figure 6 shows the dependence of the shear and fatigue resistance of the bitumen binders with the addition of polymers and coke micropowders on temperature after short-term and long-term aging. After short-term aging, the shear resistance parameter G*/sinδ of the modified binders is consistently higher than that of the original bitumen, indicating better resistance to rutting during the mixture preparation and placement stage. The fatigue failure parameter G*⋅sinδ increases with decreasing temperature. The curves for the modified bitumens are located above those of the base bitumen, with the composition with the addition of 0.1% Elvaloy and 1% coal coke micropowder (curve 5) demonstrating the highest stiffness values.

**Figure 7:**
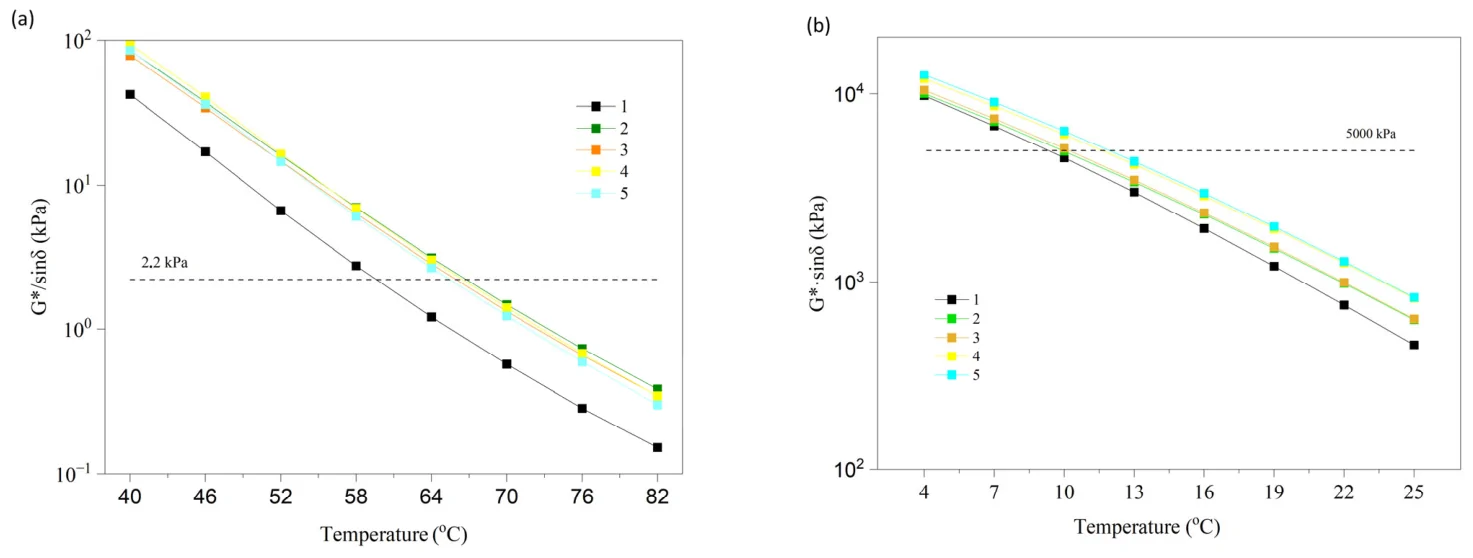
Dependences of shear (**a**) and fatigue (**b**) resistance of bitumen binders after short-term and long-term aging on temperature: 1—BND 100/130 bitumen, 2—with the addition of 0.5% Elvaloy and 0.5% coal coke nanopowder, 3—with the addition of 0.5% Elvaloy and 0.5% petroleum coke nanopowder, 4—with the addition of 0.5% SBS and 0.5% coal coke nanopowder, 5—with the addition of 0.5% SBS and 0.5% petroleum coke nanopowder.

Figure 7 shows the shear and fatigue resistance of bitumen binders with added polymers and coke nanopowders after short-term and long-term aging as a function of temperature. Nanomodified bitumens demonstrate even higher shear resistance after RTFOT aging compared to their micromodified counterparts. The fatigue properties of the nanocomposites are similar to those of the microcomposites, but the curves are shifted more uniformly, and the composition with 0.5% SBS and 0.5% petroleum coke nanopowder (curve 5) exhibits slightly higher stiffness at low temperatures.

Thus, SBS and Elvaloy polymers work synergistically with carbon fillers. Increasing the SBS content to 0.5% in nanostructured systems yields a superior shear stability at high temperatures (before aging and after RTFOT) without critically degrading the low-temperature properties.

### 3.5. Asphalt Concrete Mixtures’ Conventional Properties

The performance of the corresponding asphalt concrete mixtures was assessed through compressive strength tests at 20 °C and 50 °C and crack resistance measurements at 0 °C. These properties provide important indicators of the pavement load-bearing capacity under normal service conditions, resistance to rutting at elevated temperatures, and durability against low-temperature cracking. The incorporation of coke particles and polymer modifiers significantly influenced the mechanical performance of the asphalt mixtures by altering the internal structure and stress-transfer capability of the modified binder.

The results in Table 6 demonstrate that both the coke type and particle size affected the compressive strength of the asphalt concrete mixtures. Among the microdispersed systems, the highest compressive strength at 20 °C was obtained for the mixture containing 1 wt.% coal coke and 0.1 wt.% SBS polymer, reaching 3.29 MPa, which exceeded the minimum requirement of ST RK 1225-2019 (2.5 MPa) by approximately 32%. In comparison, mixtures containing micro petroleum coke exhibited lower strengths of 2.34–2.60 MPa. The superior compressive strength of the coal coke-modified mixtures is consistent with the surface morphology described in Section 3.1: the irregular angular shape and highly porous fractured surface of coal coke (Figure 2a) provide a larger effective contact area and better mechanical interlocking with both the binder and the aggregate skeleton than the smoother layered morphology of petroleum coke. This roughness increases the frictional resistance at the coke–bitumen interface, restricts the mobility of the asphaltene and maltene fractions, and promotes a more rigid internal binder structure. When incorporated into the mixture, this stiffened binder transfers load more effectively between aggregate particles, increasing the overall resistance to compressive deformation—consistent with prior findings that surface roughness and angularity govern the reinforcing efficiency of carbonaceous fillers in asphalt systems [40]. Similar improvements in the compressive strength resulting from the incorporation of reinforcing modifiers have been reported for polymer-modified asphalt mixtures [41].

The highest compressive strength was achieved by the mixture containing 0.5 wt.% petroleum coke nanopowder and 0.5 wt.% Elvaloy, reaching 2.62 MPa, while the corresponding coal coke formulation attained only 2.49 MPa (Table 7). Although the maximum strength of the nanocoke systems was lower than that of the best micro coal coke mixture, the nanomodified mixtures displayed more consistent performance and generally satisfied specification requirements. The improved behavior of petroleum coke nanoparticles can be attributed to their enhanced dispersion and larger interfacial surface area, which improve the stress transfer throughout the asphalt matrix [42]. Similar observations regarding nanoparticle-reinforced asphalt mixtures have been reported in recent studies on modified high-performance binders [43].

The compressive strength measured at 50 °C provides an indication of the rutting resistance and thermal stability under summer service conditions. Among the microdispersed systems, the highest value was obtained for the mixture containing micro coal coke and SBS polymer (0.80 MPa), followed closely by micro petroleum coke with Elvaloy (0.79 MPa). For the nanodispersed systems, the optimum performance was observed for petroleum coke nanopowder with Elvaloy, which achieved a strength of 0.90 MPa, representing an increase of approximately 14% compared with the best microcoke formulation.

Although modification significantly improved the high-temperature strength, all mixtures remained below the ST RK 1225-2019 requirement of 1.1 MPa for Type A and 1.3 MPa for Type B asphalt concrete. This indicates that binder modification alone was insufficient to fully satisfy the high-temperature performance requirements. The relatively low values may be associated with non-optimized binder content, aggregate gradation, or insufficient polymer dosage for the selected mixture design. Previous studies have shown that rutting resistance is highly dependent on the combined optimization of aggregate skeleton structure, binder composition, and modifier concentration rather than on binder modification alone [44]. Similar limitations have also been reported for polymer-modified asphalt mixtures when aggregate interlocking and volumetric characteristics were not simultaneously optimized [45]. Nevertheless, the higher strengths observed for Elvaloy-modified nanocoke systems indicate improved resistance to deformation at elevated temperatures and suggest the potential for further optimization [46].

The crack resistance results clearly demonstrate the advantages of nanodispersed coke systems over microdispersed modifiers. The maximum crack resistance among the microcoke mixtures was 3.53 MPa, achieved by the combination of micro petroleum coke and Elvaloy polymer, which only marginally satisfied the specification limit. In contrast, the nanocoke-modified mixtures exhibited significantly higher values ranging from 3.79 to 4.39 MPa, with the highest performance obtained for 0.5 wt.% coal coke nanopowder and 0.5 wt.% Elvaloy polymer (4.39 MPa). This value represents an improvement of approximately 24% compared with the best microcoke formulation.

## 4. Discussion

According to the comparison of the four modifiers, the particle size reduction of coke from micrometer level to nanometer level has a strong influence on the interaction mechanism of coke particles and bitumen. Microcoke is mainly utilized as a stiffening agent by providing particle reinforcement and limiting molecular movement. Nanocoke is able to reinforce due to the filler effect as well as due to the interfacial strengthening mechanisms that are induced by its high specific surface area. Due to the increased surface area of the particles, more stress transfer efficiency is also achieved, leading to the development of a more homogeneous binder structure [12].

The effectiveness of coke particles is further enhanced when combined with polymer modifiers such as SBS and Elvaloy. The rough and porous surfaces of coal coke particles facilitate polymer adsorption and physical anchoring within the asphalt matrix, while petroleum coke platelets provide extended surfaces for polymer attachment and network development. Previous morphological studies of polymer-modified bitumen have demonstrated that a well-developed polymer network significantly improves the elasticity and resistance to rutting by stabilizing the dispersed phase and reducing phase separation [47]. In the present study, the combined use of coke particles and polymers produced synergistic effects, where coke provided structural reinforcement and polymers supplied elastic recovery.

The reason why coal coke can form a higher carbon content, more developed porous structure, and higher surface activity than petroleum coke is that it has a higher carbon content and surface activity. These properties contribute to better physical contact between the coke particles and the maltene fraction of bitumen, which limits the mobility of the binder molecules, thus hardening its consistency. In previous studies, a similar rise in stiffness and decrease in softening point after the addition of carbonaceous fillers and polymer modifiers has been reported, and the particles were considered as reinforcing materials in the bitumen matrix to increase resistance to flow and permanent deformation [48,49].

The superior low-temperature performance of nanocoke systems can be attributed to their ability to provide more uniform reinforcement throughout the binder matrix. Due to their significantly smaller size and larger specific surface area, nanoparticles interact more effectively with asphalt components, promoting homogeneous stress distribution and reducing the local stress concentrations that initiate cracking [42]. Furthermore, nanoparticles improve interfacial bonding and facilitate energy dissipation during crack propagation, resulting in enhanced fracture resistance [50].

Elvaloy-modified mixtures consistently exhibited higher crack resistance than SBS-modified mixtures in both micro- and nanoscale systems. This behavior is associated with the reactive nature of Elvaloy, which improves the compatibility between the modifier and bitumen, producing a more stable and flexible internal structure capable of accommodating thermal stresses [46]. Similar improvements in low-temperature fracture resistance have been reported for polymer-modified asphalt systems containing chemically interactive modifiers [51]. The results therefore indicate that the combination of nanocoke and Elvaloy provides the most effective balance between stiffness and flexibility, leading to superior resistance against crack initiation and propagation.

A comparative evaluation of the rheological and mechanical performance in Table 8 indicates clear differences between the microdispersed and nanodispersed coke modifiers. The enhanced performance of nanocoke is primarily attributed to its smaller particle size, larger specific surface area, improved dispersion within the bitumen matrix, and more efficient stress transfer mechanisms [52]. The higher interfacial contact between nanoparticles and asphalt components promotes uniform reinforcement and reduces localized stress concentrations, resulting in improved resistance to crack initiation and propagation [53].

From both binder rheology and asphalt mixture performance perspectives, the nanocoke–Elvaloy systems served to provide the optimal balance of stiffness and flexibility, with the added benefit of superior performance in low-temperature crack resistance and more uniform stress distribution. This balanced performance is especially desired for long term pavement durability under different climatic and traffic conditions [54].

The use of coke-derived micro- and nanoparticles in combination with polymer modifiers is a good method to improve the properties of asphalt binders and asphalt concrete mixtures. The combination of microdispersed coal coke and SBS polymer gives maximum stiffness and deformation resistance, while the combination of nanodispersed coal coke, especially with Elvaloy polymer, improves the crack resistance and the stiffness/deflection balance. The results show that particle-size reduction from micro to nanoscales greatly enhances the efficiency of modifiers by improving dispersion, increasing the area of interfaces, and providing more uniform stress distribution across the entire asphalt matrix. Therefore, nanocoke–polymer systems appear to be the most promising option for the development of durable and high-performance asphalt pavements. The storage stability of the prepared binders was not evaluated in the present work because all rheological and mechanical tests were performed shortly after binder preparation. Evaluation of long-term storage stability will be considered in future studies.

### Limitations and Future Perspectives

Despite the promising performance of the developed coke/polymer-modified binders, several limitations should be acknowledged. The present study was conducted using a single base bitumen source and a limited range of coke and polymer dosages. In addition, the long-term storage stability and aging behavior of the modified binders were not evaluated. The asphalt-mixture results also indicate that binder modification alone may not be sufficient to meet all high-temperature performance requirements; therefore, aggregate gradation and binder content should be considered together with binder modification in future mixture optimization. Future studies should investigate the long-term aging and storage stability of the hybrid systems, optimize the aggregate skeleton and binder content, and extend the comparative analysis to additional coke sources and polymer chemistries. Such studies would help establish more general structure–property relationships and facilitate the practical application of coke/polymer hybrid modification in durable asphalt pavements.

## 5. Conclusions

The successful hybrid modification of asphalt binders by mixing micro and nanodispersed petroleum and coal coke, SBS and Elvaloy polymers was investigated in this study. The developed modification method improved the rheological properties of polymer–bitumen binders and the mechanical properties of asphalt concrete mixtures. The main conclusions are as follows:

❖ Coal coke exhibited a stronger stiffening effect than petroleum coke at the microscale, resulting in higher softening temperature and lower penetration when incorporated with SBS. This indicates that coal coke provides more effective reinforcement of the bitumen matrix than petroleum coke under comparable modification conditions.
❖ SBS provided higher stiffening and rutting resistance than Elvaloy at comparable coke contents, as evidenced by its lower phase angle and higher complex shear modulus. This behavior is attributed to the formation of a stronger elastic polymer network within the binder.
❖ Reducing the coke particle size from the microscale to the nanoscale reduced the performance differences between coal and petroleum coke and promoted a more uniform modification effect. The nanodispersed systems also exhibited improved resistance to cracking, suggesting more effective stress distribution within the modified binder matrix.
❖ Microcoke–SBS systems were more effective for maximizing the stiffness, high-temperature performance, and rutting resistance, whereas nanocoke–Elvaloy systems provided a more favorable balance between elasticity, thermal stability, and crack resistance. The different performance trends demonstrate that the optimum modifier combination depends on the targeted pavement performance.
❖ At the mixture level, microcoke–SBS provided the highest compressive-strength improvement at moderate temperature, whereas nanocoke–Elvaloy showed superior performance at elevated temperature and higher crack resistance.

The results indicate that microcoke is a potent reinforcing agent for bituminous binders and that SBS is even more effective in enhancing the stiffness and rutting resistance of the binder by creating a strong elastic polymer network. Both microcoke and nanocoke modifications improved the performance, but in both cases, the nanocoke-modified system showed better crack resistance and a more uniform mechanical response. The mixtures with nanodispersed coke and Elvaloy polymer resulted in the highest crack resistance, indicating the effectiveness of the nanoreinforcement for uniform stress distribution and reduction of crack propagation.

## Figures and Tables

**Table 1:** Physico-chemical properties of petroleum and coal coke samples.

Characteristics	Petroleum Coke	Coal Coke
Total moisture content, wt.%	0.2	6.9
Volatile matter content, wt.%	7.6	9.2
Ash content, wt.%	0.1	2.9
Sulfur content, wt.%	3.9	1.1
Heat of combustion, MJ/kg	36.5	31.0

**Table 2:** Physico-mechanical properties of BND 100/130 bitumen.

Characteristics	BND 100/130
Penetration at 25 °C, 0.1 mm	113.0
Penetration at 0 °C, 0.1 mm	32.0
Softening point, °C	44.0
Extensibility at 25 °C, cm	>150
Extensibility at 0 °C, cm	6.9
Dynamic viscosity at 60 °C, Pa⋅s	138.0
Dynamic viscosity at 135 °C, mm^2^/s	352.0
Flash point, °C	282.0
Fraass brittleness temperature, °C	−24.0
Solubility, %	99.9
Paraffin content, %	0.4

**Table 3:** Properties of polymers.

Indicator	SBS	Elvaloy
Density at 20 °C, g/cm^3^	0.94	0.94
Melt index at 190 °C for 2.16 kg	0.8	8.0
Volatile matter content, wt.%	0.04	0.01
Ash content, wt.%	0.2	0.1
Maximum operating temperature, °C	260.0	280.0

**Table 4:** Physico-mechanical characteristics of polymer–bitumen binders modified with microdispersed coke samples.

Polymer Content, wt.%		Penetration at 25 °C,	Softening Point,	Bitumen
SBS	Elvaloy	0.1 mm	°C	Grade
with 1 wt.% of petroleum coke				
0.1	−	70.7	66.2	BMP 70/100
0.5	−	57.7	53.1	−
1	−	61.0	53.0	−
−	0.1	76.7	66.5	BMP 70/100
−	0.5	57.3	54.9	−
−	1	60.0	53.8	−
with 1 wt.% of coal coke				
0.1	−	22.3	73.7	−
0.5	−	35.0	65.2	BMP 35/50
1	−	68.0	51.1	−
−	0.1	60.3	52.4	−
−	0.5	69.3	68.1	BMP 50/70
−	1	65.0	53.0	−

**Table 5:** Physico-mechanical characteristics of polymer–bitumen binders modified with nanodispersed coke samples.

Polymer Content, wt.%		Penetration at 25 °C,	Softening Point,	Bitumen
SBS	Elvaloy	0.1 mm	°C	Grade
with 0.1 wt.% of petroleum coke				
0.1	−	68.3	50.6	−
0.5	−	66.0	48.9	−
1	−	63.7	50.5	−
−	0.1	69.0	51.4	−
−	0.5	66.3	52.7	−
−	1	62.0	53.1	−
with 0.5 wt.% of petroleum coke				
0.5	−	70.0	62.0	BMP 70/100
−	0.5	70.0	62.3	BMP 70/100
with 0.5 wt.% of coal coke				
0.5	−	71.0	63.0	BMP 70/100
−	0.5	71.0	63.1	BMP 70/100
−	1.0	68.3	52.6	−

**Table 6:** Test results of modified asphalt concrete mixtures filled with microdispersed coke containing polymer–bitumen binders.

Modifier and Its Content, Mass %				Density, g/cm^3^	Compressive Strength at 20 °C, MPa	Compressive Strength at 50 °C, MPa	Crack Resistance at 0 °C, MPa
Petroleum Coke	Coal Coke	Polymer SBS	Polymer Elvaloy				
1	−	0.1	−	2.22	2.34	0.50	2.44
1	−	−	0.1	2.19	2.60	0.79	3.53
−	1	0.1	−	2.19	3.29	0.80	3.35
−	1	−	0.1	2.21	2.88	0.66	3.35
Requirement of ST RK 1225-2019 for type A				−	not less than 2.5	not less than 1.1	3.5–6.5
Requirement of ST RK 1225-2019 for type B				−	not less than 2.5	not less than 1.3	3.5–6.5

**Table 7:** Test results of modified asphalt concrete mixtures filled with nanodispersed coke containing polymer–bitumen binders.

Modifier and Its Content, Mass %				Density, g/cm^3^	Compressive Strength at 20 °C, MPa	Compressive Strength at 50 °C, MPa	Crack Resistance at 0 °C, MPa
Petroleum Coke	Coal Coke	Polymer SBS	Polymer Elvaloy				
0.5	−	0.5	−	2.20	2.52	0.81	3.79
0.5	−	−	0.5	2.22	2.62	0.90	4.17
−	0.5	0.5	−	2.17	1.94	0.52	3.41
−	0.5	−	0.5	2.21	2.49	0.86	4.39
Requirement of ST RK 1225-2019 for type A				−	not less than 2.5	not less than 1.1	3.5–6.5
Requirement of ST RK 1225-2019 for type B				−	not less than 2.5	not less than 1.3	3.5–6.5

**Table 8:** Comparison of the rheological characteristics and asphalt mixture performance parameters of microdispersed and nanodispersed coke–polymer-modified systems.

No.	Property	Best Microcoke System	Best Nanocoke System	Performance Advantage
1	Modifier composition	1 wt.% coal coke + 0.5 wt.% SBS	0.5 wt.% petroleum coke + 0.5 wt.% Elvaloy	Higher performance
2	Complex shear modulus, |G*|	~10^5^ Pa	~10^4^–10^5^ Pa	Microcoke provides higher stiffness
3	Minimum phase angle (δ)	55–65°	<55°	Nanocoke provides higher elasticity
4	Compressive strength at 20 °C (MPa)	3.29	2.62	Microcoke + SBS (+25.6%)
5	Compressive strength at 50 °C (MPa)	0.8	0.9	Nanocoke + Elvaloy (+12.5%)
6	Crack resistance at 0 °C (MPa)	3.53	4.39	Nanocoke + Elvaloy (+24.4%)
7	Compliance with crack resistance requirement	Meets lower limit	Fully satisfies requirement	Nanocoke superior
8	Dispersion efficiency	Moderate	High	Nanocoke superior

## Data Availability

The data that support the findings of this study are included within this manuscript.
